# Biological lignocellulose solubilization: comparative evaluation of biocatalysts and enhancement via cotreatment

**DOI:** 10.1186/s13068-015-0412-y

**Published:** 2016-01-12

**Authors:** Julie M. D. Paye, Anna Guseva, Sarah K. Hammer, Erica Gjersing, Mark F. Davis, Brian H. Davison, Jessica Olstad, Bryon S. Donohoe, Thanh Yen Nguyen, Charles E. Wyman, Sivakumar Pattathil, Michael G. Hahn, Lee R. Lynd

**Affiliations:** Thayer School of Engineering, Dartmouth College, 14 Engineering Drive, Hanover, NH 03755 USA; BioEnergy Science Center Oak Ridge National Laboratory Oak Ridge, Oak Ridge, TN USA; National Renewable Energy Laboratory, 15013 Denver West Parkway, Golden, CO 80401 USA; Biosciences Division, Oak Ridge National Laboratory, Oak Ridge, TN 37831 USA; Center for Environmental Research and Technology (CE-CERT), Bourns College of Engineering, University of California, Riverside, 1084 Columbia Ave, Riverside, CA USA; Complex Carbohydrate Research Center, The University of Georgia, 315 Riverbend Road, Athens GA, 30602 USA

**Keywords:** Biological solubilization, Lignocellulose, Cotreatment

## Abstract

**Background:**

Feedstock recalcitrance is the most important barrier impeding cost-effective production of cellulosic biofuels. Pioneer commercial cellulosic ethanol facilities employ thermochemical pretreatment and addition of fungal cellulase, reflecting the main research emphasis in the field. However, it has been suggested that it may be possible to process cellulosic biomass without thermochemical pretreatment using thermophilic, cellulolytic bacteria. To further explore this idea, we examine the ability of various biocatalysts to solubilize autoclaved but otherwise unpretreated cellulosic biomass under controlled but not industrial conditions.

**Results:**

Carbohydrate solubilization of mid-season harvested switchgrass after 5 days ranged from 24 % for *Caldicellulosiruptor bescii* to 65 % for *Clostridium thermocellum*, with intermediate values for a thermophilic horse manure enrichment, *Clostridium clariflavum, Clostridium cellulolyticum*, and simultaneous saccharification and fermentation (SSF) featuring a fungal cellulase cocktail and yeast. Under a variety of conditions, solubilization yields were about twice as high for *C. thermocellum* compared to fungal cellulase. Solubilization of mid-season harvested switchgrass was about twice that of senescent switchgrass. Lower yields and greater dependence on particle size were observed for *Populus* as compared to switchgrass. Trends observed from data drawn from six conversion systems and three substrates, including both time course and end-point data, were (1) equal fractional solubilization of glucan and xylan, (2) no biological solubilization of the non-carbohydrate fraction of biomass, and (3) higher solubilization for three of the four bacterial cultures tested as compared to the fungal cellulase system. Brief (5 min) ball milling of solids remaining after fermentation of senescent switchgrass by *C. thermocellum* nearly doubled carbohydrate solubilization upon reinnoculation as compared to a control without milling. Greater particle size reduction and solubilization were observed for milling of partially fermented solids than for unfermented solids. Physical disruption of cellulosic feedstocks after initiation of fermentation, termed cotreatment, warrants further study.

**Conclusions:**

While the ability to achieve significant solubilization of minimally pretreated switchgrass is widespread, a fivefold difference between the most and least effective biocatalyst—feedstock combinations was observed. Starting with nature’s best biomass-solubilizing systems may enable a reduction in the amount of non-biological processing required, and in particular substitution of cotreatment for pretreatment.

**Electronic supplementary material:**

The online version of this article (doi:10.1186/s13068-015-0412-y) contains supplementary material, which is available to authorized users.

## Background

Biologically mediated processing of cellulosic biomass is a promising route to sustainable production of fuels and chemicals, but requires improved approaches to producing soluble intermediates from this recalcitrant feedstock. Such approaches have been a topic of intense activity since studies were initiated in the 1940s in the US Army Natick lab [1]. Early pioneers in the field noted that although many microorganisms grow quite rapidly on cellulose, only a few produce extracellular cellulases capable of converting crystalline cellulose to glucose in vitro [2]. The aerobic fungus *Trichoderma viride* (subsequently renamed *T. reesei)* was chosen as a model organism based on its excellent enzyme secretion properties and is the industry standard today [3].

In 1980, Knappert et al. reported nearly complete solubilization of hardwood flour by *T. reesei* cellulase following pretreatment with dilute acid at elevated temperatures [4]. By the late 80s, hundreds of papers on hydrolysis of wood using fungal cellulase and various thermochemical pretreatments had appeared in the literature. Also at this time, Lynd and Grethlein showed that hydrolysis of dilute acid pretreated hardwood with cell-free broth from the thermophilic anaerobic bacterium *Clostridium thermocellum* was similar to previous reports with *T. reesei* cellulase [5]. As with the fungal system, hydrolysis yields for unpretreated controls were <20 %. Interest developed in herbaceous feedstocks, which led the Department of Energy to choose switchgrass (*Panicum virgatum*) as a model herbaceous feedstock [6]. High-yielding hydrolysis using fungal cellulases has been reported for pretreated senescent switchgrass as well as other grasses [7, 8]. An extensive literature exists on forage digestibility in ruminants, with grass digestibility inversely related to harvest age (reviewed in [9]) and generally greater than digestibility of wood [10]. Solubilization is thought to be enhanced by the alternating microbial solubilization and mechanical disruption that occurs during rumination [11, 12].

Enzymatic hydrolysis with simultaneous wet milling has been investigated as an alternative to thermochemical pretreatment, with most reports focusing on hydrolysis of newsprint or wood by fungal cellulases. Kelsey and Shafizadeh achieved 4- to 5-fold higher sugar yields with milling media as compared to unmilled controls and twofold higher yields when milling was done during hydrolysis rather than beforehand [13]. This approach was investigated further in “attrition bioreactors”, with several studies showing that continuous ball milling or high shear during enzymatic hydrolysis enabled a roughly twofold increase in hydrolysis yields [14–17]. More recently, attrition milling of thermochemically pretreated substrates has been used as a means of reducing required enzyme loadings [18, 19], and long periods of ball milling have been shown to increase enzymatic hydrolysis of maize [18, 20]. Continuous wet milling has also been shown to enhance anaerobic digestion [21] by disrupting floc structure and cell walls. There have been few published reports investigating milling in the context of microbial processing of cellulosic biomass to liquid fuels, and none to our knowledge that evaluated milling of partially converted feedstock.

Considerable literature exists on the comparative performance of various pretreatment processes and feedstocks with respect to enzymatic hydrolysis using fungal cellulase [7, 8, 22]. Comparative studies of minimally fractionated cell-free cellulase preparations originating from different microorganisms have also been reported, including several studies which found cellulases from *C. thermocellum* to have higher specific activity than fungal cellulases on model substrates [23, 24]. In order to achieve high solubilization of glucan in ammonia-soaked rice straw, Waeonukul et al. found tenfold less β-glucosidase-supplemented cellulase was required for *C. thermocellum* cellulase in comparison to a commercial cellulase from *T. reesei* (Celluclast 1.5) [25]. Kanafuse-Shinkai et al. found that sugar release from Avicel, unpretreated Timothy and rice straw was about two times higher for cellulase prepared from the anaerobic extreme thermophile *Caldicellulosiruptor bescii* as compared to a *T. reesei* cellulase preparation [26]. In 2009, Yang et al. reported significant solubilization of both woody (*Populus*) and herbaceous (switchgrass) feedstock without pretreatment or added enzymes by cultures of *C. bescii* [27]. In a subsequent study, Kataeva et al. [28] reported equal fractional solubilization of lignin and carbohydrate components during fermentation of washed unautoclaved senescent switchgrass by *C. bescii*. Enhanced effectiveness of cellulose solubilization by microbial cultures compared to cell-free enzyme preparations has also been noted [29, 30]. Naturally cellulolytic microbes could potentially be used to convert cellulosic biomass into fuels without added enzymes, an approach offering both large advantages and substantial challenges compared to the more conventional approach of processes featuring dedicated cellulase production [31].

There are few reports of controlled comparisons of lignocellulose solubilization by microbial cultures. Yee et al. showed that fermentation of dilute acid treated switchgrass by *C. thermocellum, C. bescii,* and *Caldicellulosiruptor obsidiansis* results in higher concentrations of fermentation products as compared to simultaneous saccharification with fungal cellulase and yeast [32] (subsequently referred to as SSF). Shao et al. show solubilization of autoclaved but otherwise unpretreated winter rye by *C. thermocellum* was significantly higher than by SSF [33]. In a separate study, Shao et al. found solubilization of AFEX pretreated corn stover to be comparable for *C. thermocellum* and SSF [34]. Izquierdo et al. found substantial solubilization of autoclaved but otherwise unpretreated senescent switchgrass by *Clostridium clariflavum* and *C. thermocellum*, with the highest solubilization achieved by one of the two *C. clariflavum* strains [35].

Here we examine (a) the extent to which the ability to solubilize cellulosic substrates with no pretreatment other than autoclaving (referred hereafter as “minimal pretreatment”) is widespread among different microorganisms; (b) the impact of choice of substrate, biocatalyst, and particle size on solubilization of minimally pretreated lignocellulose, and (c) augmentation of biologically mediated solubilization by mechanical disruption after partial fermentation.

## Results

### Comparative solubilization tests

Comparative tests were conducted under controlled conditions—with respect to substrate, particle size, incubation time, mixing, and reaction vessel—to assess the relative ability of various biocatalysts to solubilize plant cell walls. Biocatalyst systems were chosen to represent a range of growth temperatures (mesophilic, thermophilic, extremely thermophilic), cellulase architectures (complexed, non-complexed), and mixed enrichments as well as pure cultures. In particular, solubilization of mid-season switchgrass at 100 % elongation was assessed in batch serum vials for *Caldicellulosiruptor bescii* cultivated at 75 °C; *Clostridium thermocellum*, *Clostridium clariflavum*, and an enrichment from horse manure cultivated at 60 °C; *Clostridium cellulolyticum* cultivated at 35 °C; and simultaneous saccharification and fermentation (SSF) featuring a fungal cellulase cocktail (Novozyme Ctec2 supplemented with Htec2) and *Saccharomyces cerevisiae* incubated at 37 °C. Additional experiments were conducted in the Lynd lab (Additional file 1: Table S1, ‘Lynd Lab This Study’) and compared to prior literature reports, and in the case of SSF, control experiments carried out in the Wyman lab (Additional file 1: Table S1, ‘Comparative Studies’). For each biocatalyst system investigated, there was good agreement between results obtained in the Lynd lab and results obtained by other labs (Additional file 1: Table S1). The choice of mid-season switchgrass was based on the expectation that larger differences among biocatalysts might be seen for this feedstock than a more recalcitrant feedstock, as subsequently confirmed (below). A relatively low (5 g glucan/L) substrate concentration was used to focus on the intrinsic capabilities of microbes and/or their enzymes acting on different substrates in the absence of complicating factors expected to arise at higher concentrations in industrial settings.

**Fig. 1:**
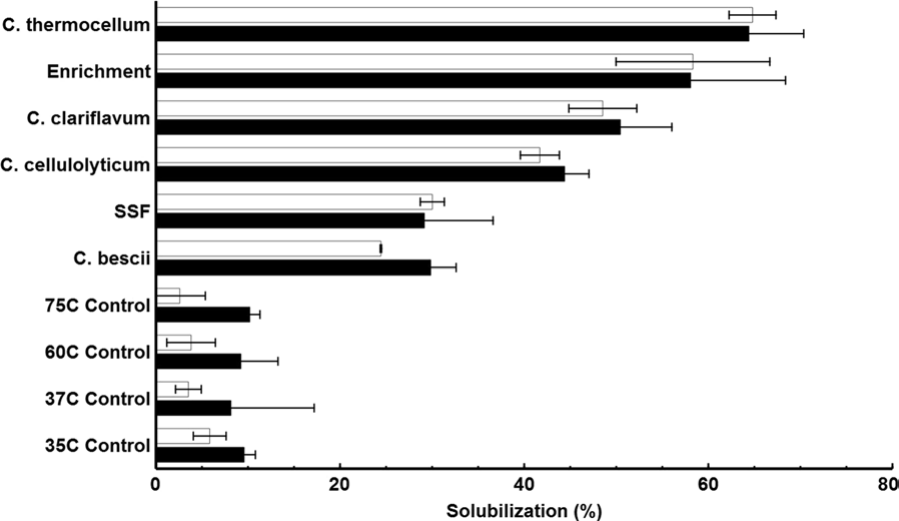
Solubilization of washed mid-season switchgrass by various biocatalysts. Xylan (*white*) and glucan (*black*) solubilization from washed mid-season switchgrass by various bacteria or SSF with yeast and fungal cellulase after 5 days. Enrichment was selected at 60 °C on Avicel from horse manure compost. Uninoculated controls (75, 60, 37 and 35 °C) for each incubation temperature were analyzed to account for non-biological solubilization. Results are expressed as mean ± SD (*n* ≥ 2)

The ability to achieve significant solubilization of minimally pretreated mid-season switchgrass was found to be widespread among the anaerobic, cellulolytic bacteria tested (Fig. 1). Respective conversion of glucan and xylan after 5 days was 65 ± 3 and 64 ± 6 % for *C. thermocellum*, 58 ± 8 and 58 ± 10 % for the horse manure enrichment, 49 ± 4 and 50 ± 6 % for *C. clariflavum*, 45 ± 2 and 47 ± 2 % for *C. cellulolyticum*, 30 ± 1 and 29 ± 7 % for SSF, and 24 ± 1 and 30 ± 3 % for *C. bescii*. Decreasing the initial substrate concentration from 5 g glucan/L to 1 g glucan/L increased glucan and xylan solubilization substantially for *C. bescii* (to 43 ± 3 and 49 ± 4 %, respectively), but not for *C. thermocellum* and SSF (Additional file 1: Figure S1). Cultures that achieved higher extents of substrate solubilization also produced higher amounts of fermentation and hydrolysis products (Additional file 1: Figure S2a). Supernatants from most bacterial cultures had a final pH below six (Additional file 1: Figure S2b) and contained significant amounts of unfermented sugars, suggesting these cultures may have become inhibited by low pH. Time course studies (Additional file 1: Figure S3A) showed that the rate of solubilization by either biocatalyst was near zero when the experiment was terminated after 5 days.

### Further comparison of *C. thermocellum* and fungal cellulase

**Fig. 2:**
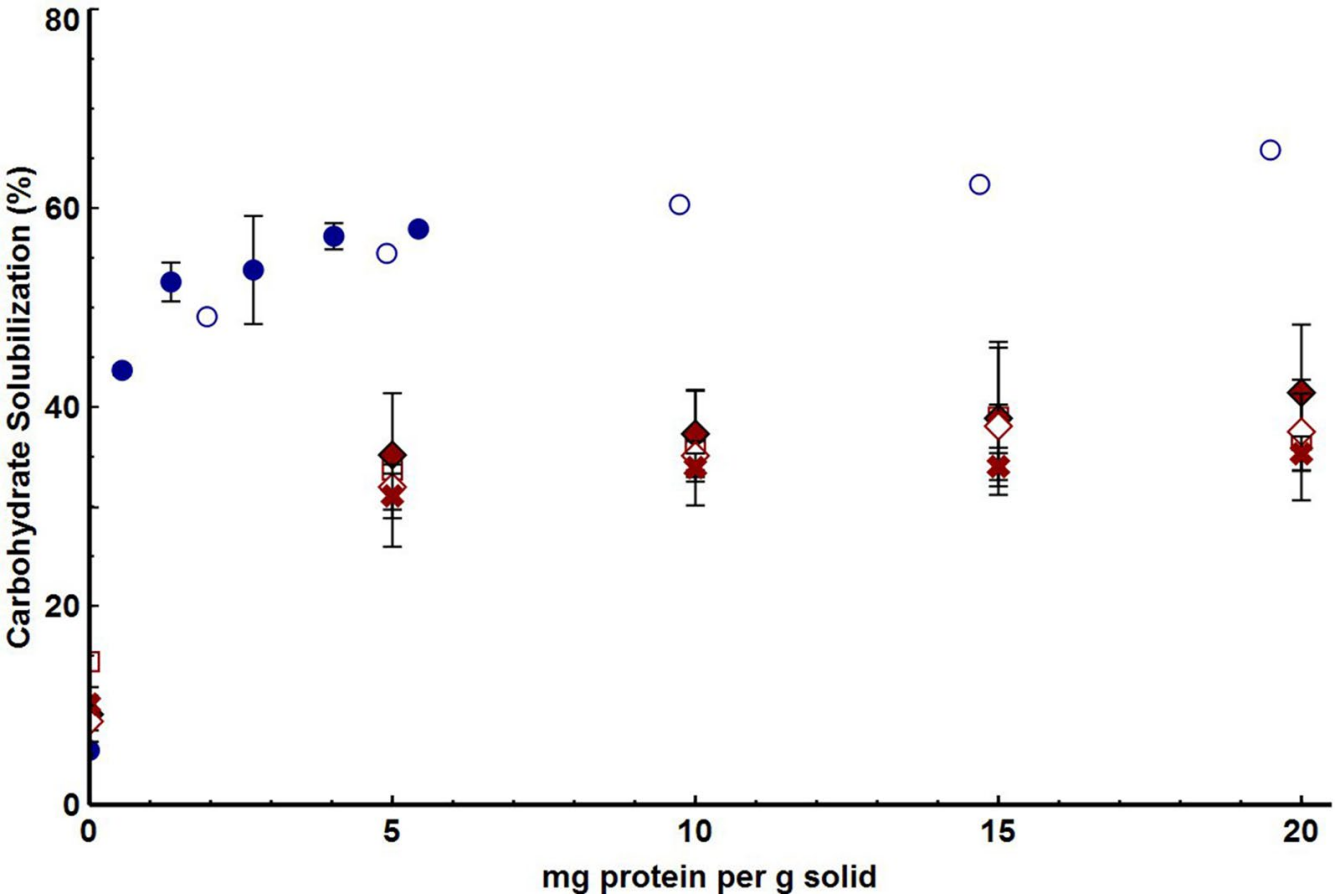
Carbohydrate solubilization from washed mid-season switchgrass by various enzyme loadings. *C. thermocellum* enzymes (*blue*) were purified by either affinity purification *filled circle* or by concentrating and dialyzing cell-free broth *circle*. Fungal cellulase (*red*) was incubated at 37C in the presence (*filled diamond*) or absence of yeast (*open diamond*), at lower substrate concentration (*X*, 1 g/L glucan, 2.5 g/L solids), or increased hydrolysis temperature (*square*, 50 °C). Results are after 5 days and are expressed as mean ± SD (*n* ≥ 2 except for dialyzed concentrated broth)

In light of the difference observed between *C. thermocellum* cultures and SSF using fungal cellulase, further comparative experiments were undertaken to assess the efficacy of these two systems under a broader range of conditions. Fractional solubilization as a function of protein loading is presented in Fig. 2. At 5 mg protein per gram substrate (total solids), *C. thermocellum* cellulosomes purified by affinity digestion or secretomes purified by concentrating and dialyzing cell-free broth solubilized nearly 60 % of the carbohydrate, with slight increases observed at increasing enzyme loading. However, overall solubilization by the fungal cellulase remained between 30 and 40 % for enzyme loadings up to 20 mg/g and was unaffected by the presence or absence of yeast or increased hydrolysis temperature. The pH did not change during the course of hydrolysis using fungal cellulase (Additional file 1: Figure S2b) and lower substrate concentration did not improve solubilization (Additional file 1: Figure S1), suggesting that hydrolysis products did not adversely affect hydrolysis.

**Fig. 3:**
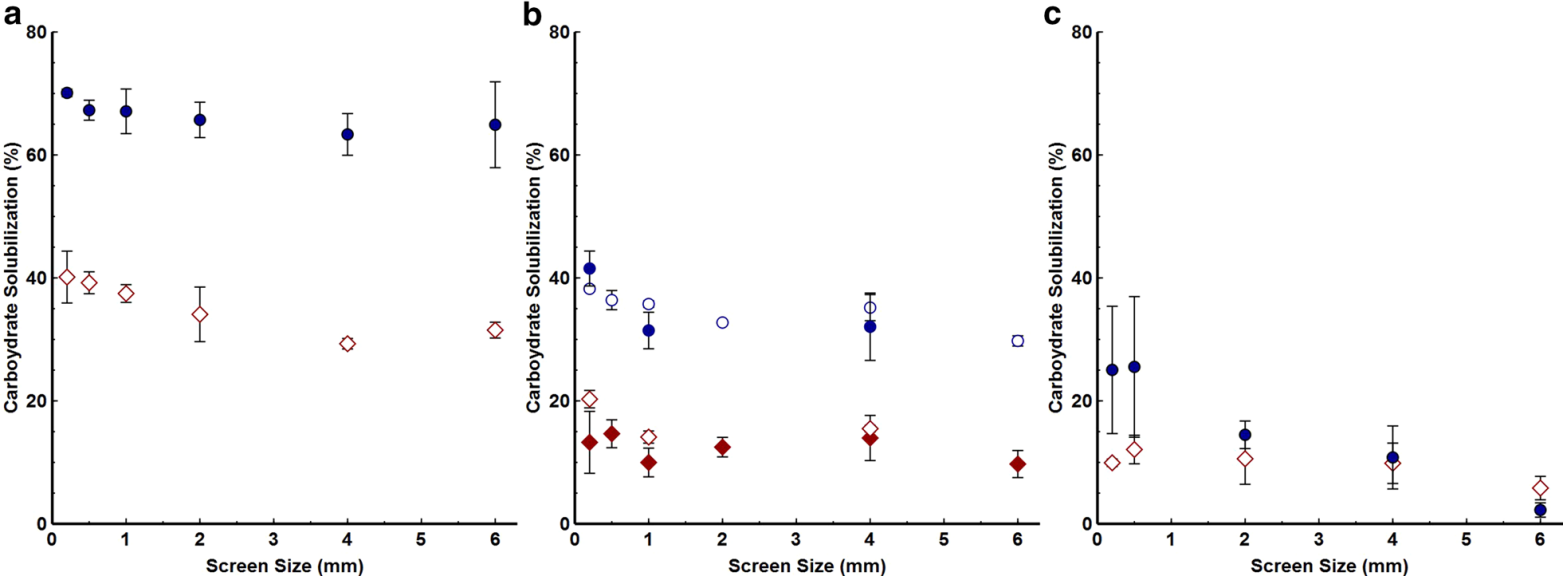
Carbohydrate solubilization by *C. thermocellum* (*circles*) or SSF (*diamonds*). Solubilization of uninoculated controls was less than 10 %. Results are expressed as mean ± SD (n ≥ 2). **a** Washed Cave in Rock switchgrass harvested in June. **b** Washed Cave in Rock switchgrass harvested in April (*filled circle*, *diamond*) or washed Alamo switchgrass harvested in October (*circle*, *filled diamond*). **c** Washed *Populus*

We next examined the impact of particle size for mid-season switchgrass as well as senescent (October harvest) switchgrass and *Populus*. Over the 30-fold range of particle sizes examined (0.2–6 mm screens), solubilization of mid-season switchgrass by *C. thermocellum* cultures was about twice that of the SSF system and decreased slightly with increasing particle size (Fig. 3a). For both biocatalysts, overall solubilization of two different senescent switchgrass samples was approximately half that of mid-season switchgrass (Fig. 3b). Solubilization of *Populus* by SSF, included in this study as a representative woody feedstock, was similar to that for senescent switchgrass and below 20 % for all particle sizes tested (Fig. 3c). *Clostridium thermocellum* fermentation of *Populus* (Fig. 3c) achieved lower solubilization than senescent switchgrass and exhibited a declining trend with increasing particle size not seen for mid-season or senescent switchgrass (Fig. 3c). Solubilization of *Populus* by SSF did not proceed after 2 days but continued to progress slowly for *C. thermocellum* (Additional file 1: Figure S3B). Considering *C. thermocellum* and SSF with minimally pretreated mid-season switchgrass, senescent switchgrass, and *Populus*, the difference in solubilization from the lowest performing biocatalyst-feedstock combination to the highest performing combination was over fivefold (Additional file 1: Figure S4).

Glycome profiling, previously used to gain mechanistic insights into biomass deconstruction [36], was applied to solids remaining after fermentation of mid-season switchgrass by *C. thermocellum* and SSF (Additional file 1: Figure S5, discussed in more detail in Appendix 2). The epitope profiles for mid-season switchgrass solubilized by both biocatalysts were very similar, despite the substantially different extent of solubilization observed and the different mechanisms employed. However, greater removal of pectic components (e.g., rhamnogalacturonan, homogalacturonan, and pectic arabinogalactan epitopes) is detected for *C. thermocellum* fermentation compared to SSF. Glycome profiles changed less for solubilization of *Populus* as compared to switchgrass for both conversion systems, presumably due to the low solubilization observed.

### Plant cell wall solubilization trends

**Fig. 4:**
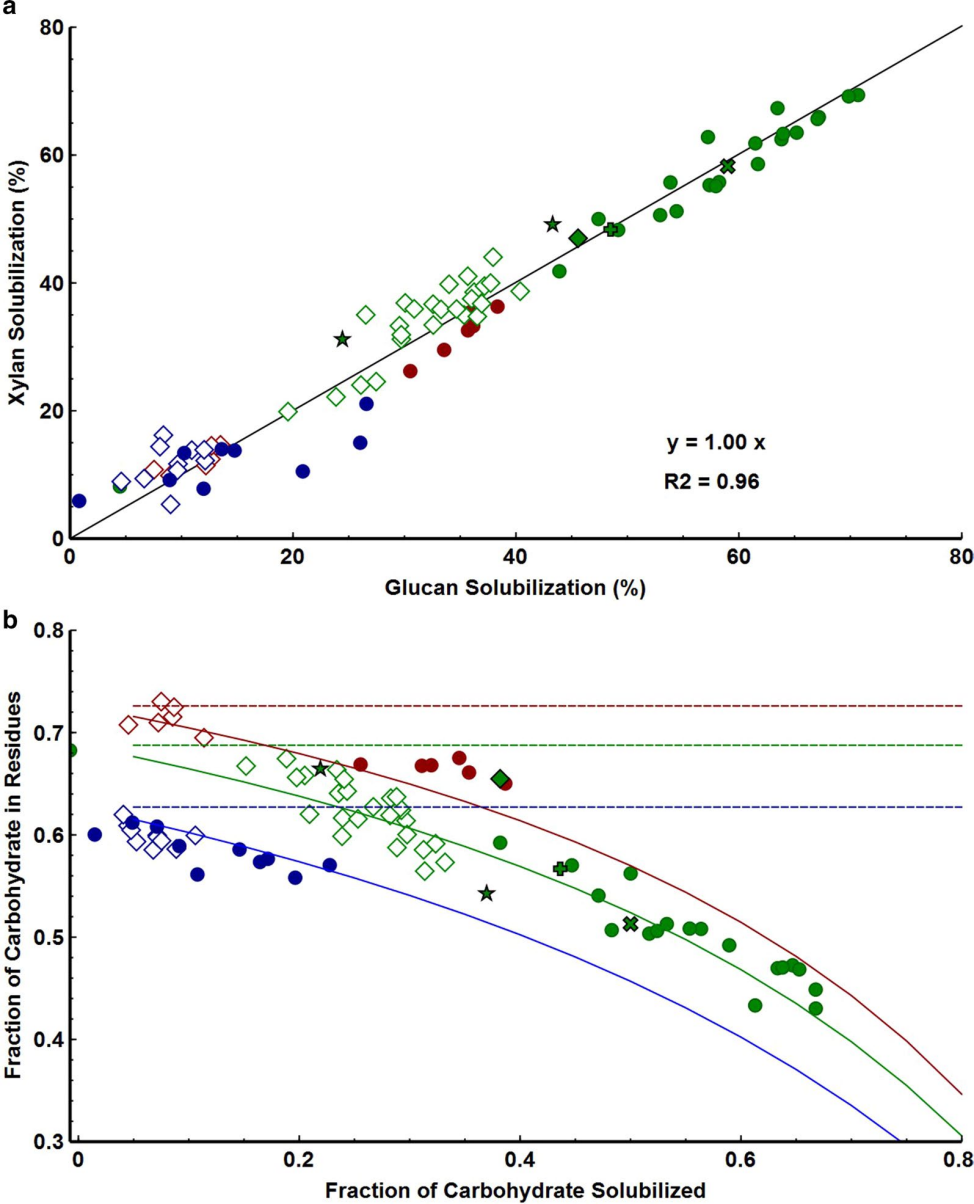
Solubilization data from three feedstocks, six biocatalysts, and including both time-course and end-point measurements. Results are expressed as mean (n ≥ 2) **a** Xylan and glucan solubilization. **b** Fractional carbohydrate content of residual solids after various extents of solubilization. Data were compared to derived equations for two hypothesis (1) Equal fractional solubilization of carbohydrate and non-carbohydrate (*dashed lines*); (2) Non-carbohydrate fraction is inert (*solid lines*). The three *lines* for each model result from different initial carbohydrate compositions of the feedstocks

We sought to test and identify general trends for plant cell wall solubilization taking into consideration data described for six different biocatalysts, three feedstocks, and various conditions in Additional file 1: Figures 1–3 S1 and S4 presented above. Fractional solubilization of xylan and fractional solubilization of glucan were equal (*r*^2^ = 0.96), as shown in Fig. 4a. We next used this data set to test two hypotheses regarding solubilization of non-carbohydrate components: (1) equal fractional biological solubilization of carbohydrate and non-carbohydrate components, and (2) non-carbohydrates remaining inert. If hypothesis 1 were correct, then the fractional carbohydrate content, *f*_*C*_, would be unchanged by reaction, or

1 $$f_{C} = {^\circ }f_{C}$$

where °*f*_*C*_ is the mass fraction of carbohydrate in solids from uninoculated controls.Results expected on this hypothesis are plotted as a function of *f*_*C*_ in Fig. 4b (dashed lines).

If the inert noncarbohydrate fraction hypothesis were correct, then it may be shown (Supplemental Appendix 1) that

2 $$f_{C} = \frac{{(1 - S_{C(0)}^{C} ){^\circ }f_{C} }}{{\left( {1 - S_{C(0)}^{C} } \right){^\circ }f_{C} + {^\circ }f_{NC} }}$$

where °*f*_*NC*_ is the mass fraction of non-carbohydrate in solids from uninoculated controls; *f*_*C*_ is the mass fraction of carbohydrate in the solids at time *t*; and $S_{C(0)}^{C}$ is the fraction of initial carbohydrate solubilized biologically.

As may be seen from Fig. 4b, a plot of *f*_*C*_ vs $S_{C(0)}^{C}$ is consistent with the inert noncarbohydrate fraction hypothesis as described by equation [2] but is not consistent with the equal fractional solubilization hypothesis.

### Enhancement of solubilization by cotreatment

**Fig. 5:**
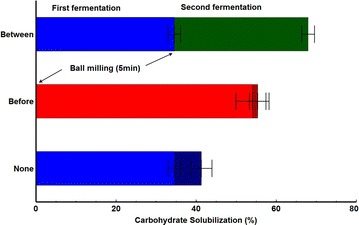
Carbohydrate solubilization of washed senescent switchgrass (Alamo) during successive 5-day fermentations. Fermentations were conducted as described in the text. *Green* milling between first and second fermentation. *Red* milling before the first fermentation. *Blue* no milling. Results are expressed as mean ± SD (*n* ≥ 2)

Mechanical disruption was investigated as a means to augment solubilization of senescent switchgrass by *C. thermocellum* cultures, either as a pretreatment (before fermentation) or as a cotreatment (after partial fermentation). Senescent switchgrass was fermented in pH controlled bioreactors in two 5-day stages. Residual solids were recovered from the first stage, washed, and wet ball milled for 5 min at approximately 13 wt.  % solids, or not milled. Solids (unmilled, or milled) were resuspended, autoclaved, reinoculated, and fermented in second stages for another 5 days. Carbohydrate solubilization during the first stage was 35 ± 2 %, consistent with results from serum vials (Fig. 3b). However, ball milling between fermentations increased solubilization during the second stage from 10 ± 2 % (without milling) to 51 ± 2 % (with milling). When the same milling was applied as a pretreatment before the first fermentation stage, carbohydrate solubilization was 54 ± 4 %, and no further solubilization occurred in the second stage. Overall solubilization for the two fermentations with ball milling between fermentation stages was significantly higher (68 ± 2 %) than control fermentations without ball milling (41 ± 2 %), or with wet ball milling before the first inoculation (55 ± 2 %) (Fig. 5).

**Fig. 6:**
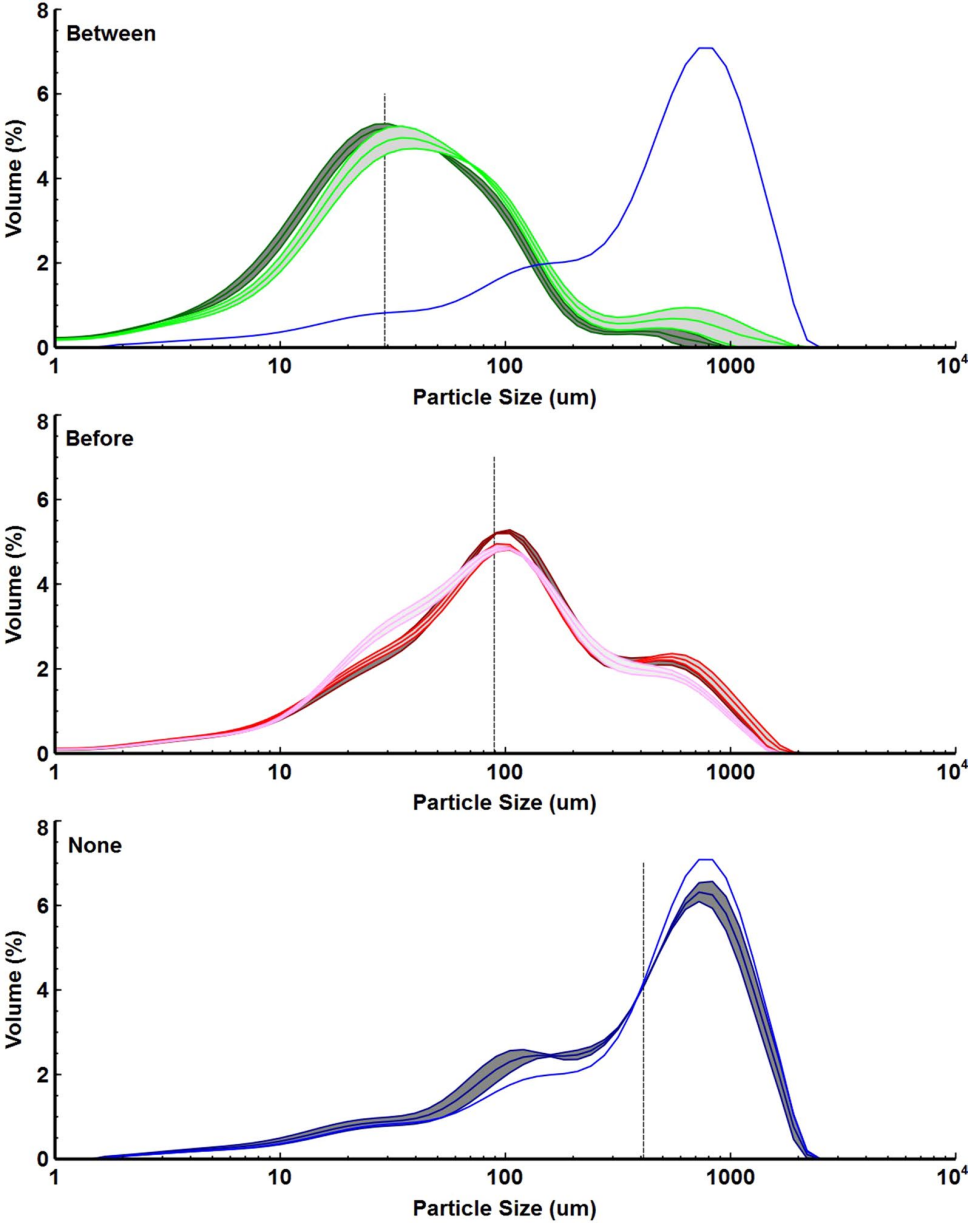
Particle size distributions. Particle size distributions of residual solids from *C. thermocellum* fermentation of washed senescent switchgrass without (*blue*) or with brief milling before (*red*) or after (*green*) partial fermentation (see Fig. 5). Particle size distribution (mean ± SD, *n* ≥ 2 except for no milling condition after 1 fermentation stage) is shown for each milling condition either after one (moderate hue) or two (darkest hue) fermentation stages. Measurement of initial partial size distribution was only possible for the milling before condition (*lightest color* hue). Volume-mean particle size after two fermentations is indicated by the dashed line for each milling condition

**Fig. 7:**
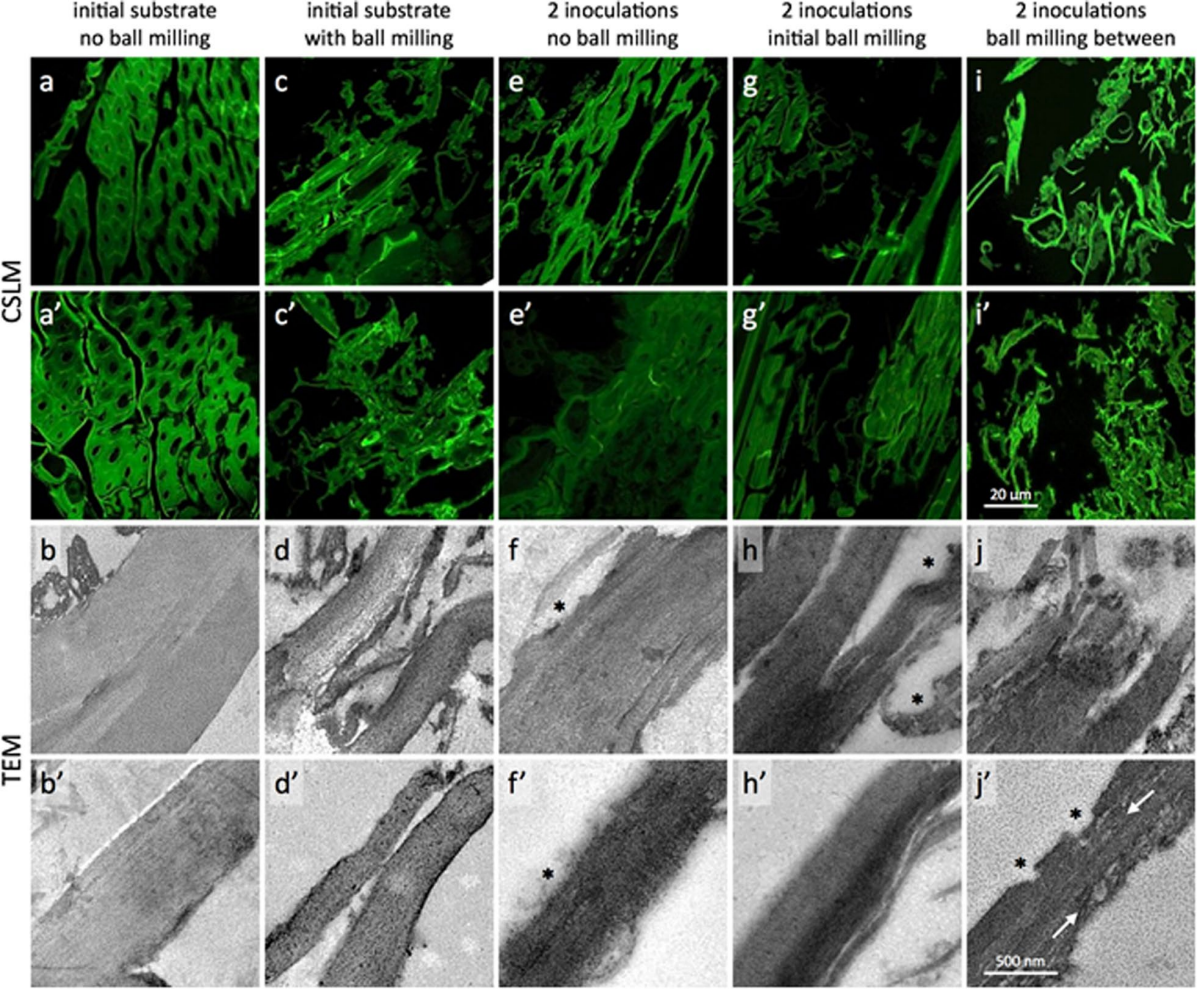
Confocal scanning laser micrographs (CSLM, *top*) and transmission electron micrographs (TEM, *bottom*) of residual solids. Switchgrass tissue samples remaining in the residues of *C. thermocellum* fermentations with and without ball milling were imaged. Some disruption at the tissue scale can be seen as dislocations among regions of fiber cells even in the initial substrate without any ball milling (**a**, **a’**). This is likely caused by the knife milling. However, the impact of the knife milling alone does not seem to propagate into the cell walls as almost no delamination or surface ablation was observed in these cell walls (**b**, **b’**). The impact of the ball milling on the initial switchgrass was evident at both the micron (**c**, **c’**) and nanometer scale (**d**, **d’**). More dislocation of the cells is apparent, but now it appears to coincide with fracturing and delamination of the cell walls. All of the inoculation residues share common features of altered cell wall textures evidenced by a hazy (**e’**, **g’**) or mottled staining density (**f’**, **j’**), and irregular, sometimes scalloped surfaces (*asterisks*, **f**, **f’**, **h**, **j’**). Clearly the most dramatic change in tissue and cell wall structure was seen in the sample that was ball milled between the two inoculations (**i**, **j**). Almost no intact tissue is visible with single cells and cell wall fragments dominant (**i**, **i’**). Also, there was more frequent evidence of void space opened up deep within the cell walls (*white arrows*, **j’**)

Residual solids from the experiment depicted in Fig. 5 were characterized to gain insight into the mechanisms underlying the effectiveness of milling. Initial ball milling reduced the particle size significantly; however, milling after the first fermentation achieved a significantly greater particle size reduction (Fig. 6). Respective final *d*_50_ (volume-mean particle size) values were 411 ± 42 µm for no milling, 89 ± 6 µm for milling before the first fermentation, and 29 ± 2 µm for milling between the first and second fermentations (Additional file 1: Table S2). Milling had little effect on substrate cellulose crystallinity (Additional file 1: Figure S6), but pore volume increased with milling and fermentation (Additional file 1: Table S2). Imaging showed that after ball milling between the first and second fermentation, almost no intact tissue is visible with single cells and cell wall fragments dominant (Fig. 7i, i′). In addition, there was more frequent evidence of void space opened up deep within the cell walls (white arrows, Fig. 7j′). This is in contrast with residues with initial ball milling (Fig. 7c, d, g, h) or unmilled control (Fig. 7a, b, e, f), which still had intact tissue after two fermentations.

## Discussion

The extent of solubilization of lignocellulosic substrates by various biocatalysts and with no pretreatment other than autoclaving was documented under controlled, but not industrial conditions, in the most comprehensive such comparative study to date. Glucan and xylan solubilization yields obtained by three bacterial cultures (*C. thermocellum*, *C. clariflavum,* and *C. cellulolyticum*) were substantially higher (Fig. 1) than the yields obtained by SSF. At an initial substrate concentration of 5 g glucan/L, the extreme thermophile *C. bescii* exhibited the lowest solubilization of the systems tested. However, at an initial glucan concentration of 1 g/L, *C. bescii* exhibited higher solubilization than the SSF, suggesting that this organism has strong intrinsic biomass solubilization capability but that growth and/or enzymes are more susceptible to inhibition. A strong correlation between the rate of microbial growth on model cellulosic substrates with increasing temperature has previously been observed [31], although conditions were not controlled. Although the highest solubilization yields observed herein were at 60° (*C. thermocellum* and the horse manure enrichment), the range of yields observed for thermophilic systems overlapped that for mesophilic systems.

The observation here of roughly twofold higher lignocellulose solubilization yields for *C. thermocellum* as compared to SSF on microporous lignocellulosic substrates is remarkable—and as yet not understood—in light of the much larger size of the *C. thermocellum* cellulosome complex as compared to the components of the non-complexed *T. reesei* cellulase system. We saw little additional solubilization in response to increased enzyme loading above 5 mg/g mid-season switchgrass for either fungal or *C. thermocellum* cellulases. Increasing hydrolysis yields from similar enzyme loadings have been seen for fungal cellulases acting on pretreated substrates [7, 8, 22], as might be expected due to increased substrate accessibility following pretreatment. Our results differ from those of Resch et al., who reported similar solubilization of minimally pretreated senescent switchgrass by equal loadings of either fungal or *C. thermocellum* cellulases [37]. This discrepancy remains to be explained.

Enhanced rates of cellulose solubilization have been observed in the presence of metabolically active microorganisms as compared to cell-free enzyme systems [29, 30]. We looked for, but did not find, increased extents of solubilization in experiments involving actively fermenting cells as compared to cell-free cellulase preparations. Adding high levels of *C. thermocellum* cellulase (Fig. 2) does not result in higher 5-day solubilization as compared to microbial cultures (Fig. 1). The results reported here do not preclude such enzyme-microbe synergy with respect to the rate of biomass solubilization.

Data drawn from three feedstocks, six biocatalysts, and including both time-course and end-point measurements exhibit a strong correlation between glucan and xylan solubilization, which are equal on a fractional basis. This is consistent with coordinated and mutually dependent solubilization of these two components, although different enzyme systems are known to be involved [31, 38]. This extensive data set is consistent with the non-carbohydrate fraction of cellulosic feedstocks being inert and is not consistent with proportional biological solubilization of carbohydrate and non-carbohydrate components. A prior report of proportional solubilization [28] was based on characterization of residual solids that would not pass through a glass filter with a pore size of 40–60 µm and included non-biological as well as biological solubilization. Although the data in Fig. 4b are for biological solubilization with subtraction of solubilization observed in uninoculated controls, the same pattern of declining residual carbohydrate fraction with increasing fractional conversion was observed whether non-biological solubilization was subtracted or not, which is inconsistent with proportional solubilization of all biomass components.

We hypothesized that glycome profiling might reveal large differences in the composition and linkages present in unsolubilized substrates in light of the different mechanisms employed by *C. thermocellum* and fungal cellulase as well as the substantially different extents of solubilization observed for these two biocatalysts. However, only small differences were in fact observed. Notable disruption of feedstock particles was observed microscopically following cultivation with *C. thermocellum* and was significantly enhanced by ball milling.

Since even the most effective systems examined here achieve less than 40 % solubilization of senescent switchgrass under favorable conditions, it is logical to look to non-biological strategies to increase solubilization. Disruption of the lignocellulose matrix prior to biological processing (pretreatment) has been investigated extensively. Considerably less attention has been given to non-biological disruption after biological attack has begun, or “cotreatment”. As noted by Weimer et al. [39], alternating mechanical and biological disruptions are important factors underlying the high solubilization of grass realized by ruminants, and this approach has promise in the context of industrial processes.

Brief ball milling of residual solids from fermentation of senescent switchgrass by *C. thermocellum* followed by a second fermentation nearly doubled overall solubilization yields as compared to that achieved after a single fermentation without ball milling. It is notable that the fractional solubilization of carbohydrate present at the start of the respective fermentation was higher for the second fermentation (51 ± 2 %) than for the first fermentation (35 ± 2 %). We saw little impact of brief milling on crystallinity, consistent with prior reports [20, 40, 41]. Our results highlight the importance of accessibility in determining hydrolysis yields. We observed that milling after partial biological solubilization was more effective at enhancing solubilization than milling prior to fermentation, as have others [14, 15], and also found that milling after partial solubilization was more effective at reducing particle size. The substantial impact of brief milling observed here supports the possibility of mechanical disruption in a vessel much smaller than the fermenter. By contrast, prior investigations of cotreatment, under various names, have generally employed continuous milling [14–16] requiring that milling occur in the hydrolysis reactor or a comparably sized vessel. Whereas we studied the impact of milling during fermentation, prior reports using milling to enhance solubilization of lignocellulose have focused on enzymatic hydrolysis in the absence of cells. Energy requirements for milling as a pretreatment for solubilization using fungal cellulase are known to prohibitively high [42, 43]. We are optimistic that energy requirements for cotreatment-enhanced thermophilic fermentation can be much lower, but leave this important topic to a future report.

## Conclusions

Our results provide directional guidance for development of advanced processes that look beyond the fungal cellulase/thermal pretreatment paradigm. Key process-relevant lessons we take from these results are as follows:

- Some biocatalysts, some feedstocks, and some biocatalyst-feedstock combinations are much more effective than others at achieving high solubilization with minimal pretreatment, with the extents of solubilization achieved by several bacterial systems substantially higher than for fungal cellulase and a fivefold difference between the most effective and least effective combinations;
- Starting with nature’s best biomass-solubilizing systems may enable a reduction in the amount of non-biological processing required, and in particular substituting cotreatment for pretreatment.

Although promising, further work is required to translate these results into industrial practice. In particular, the biocatalysts we found to be most effective at solubilizing biomass are non-model microorganisms for which limited molecular tools are available and extensive development and testing under industrial conditions are required, e.g. with respect to solids loading. In addition, optimization, innovation, and evaluation pursuant to a diversity of cotreatment strategies in conjunction with these biocatalysts have yet to be undertaken.

## Methods

### Microbes

*Clostridium thermocellum* strain DSM 1313 (DSMZ, Braunschweig, Germany), DC3 enrichment (described by Reed et al. [44]), and *C. clariflavum* DSM 19732 (DSMZ) were cultured in LC media as described previously [45] with 5 g/L MOPS and 1 g/L l-cysteine hydrochloride monohydrate at 60 °C. For experiments with *Populus*, *C. thermocellum* was cultured in MTC as previously described [45] since growth was more reliable than in LC media. *C. cellulolyticum* H10 (gift from Mascoma Corp, Lebanon NH) was cultured a modified CM3 media [46] at 35 °C and *C. bescii* DSM 6725 (gift from Dr. Michael Adams, University of Georgia, Athens) was cultured at 75 °C in modified Medium 516, as previously described [27], with the addition of 5 g/L MOPS.

### Enzymes

Cellic Ctec2 (>1000 Biomass Hydrolysis Units (BHU-2) per gram, density of 1.226 g/mL, 170 g protein/L) and Htec2 (2698.17 Fungal Xylanase Units (FXU-S) per gram, density of 1.209 g/mL, 180 g protein/L) (Novozymes, Franklinton NC) was used at 4.5 and 0.5 mg per gram solid, respectively, unless otherwise noted. Activity Measurement of solubilization of mid-season switchgrass taken at the start and end of the period over which experiments reported herein were carried out showed consistent yields. Enzymes were incubated with or without Saccharomyces cerevisiae (NREL) at either 37 or 50 °C in KN medium as described previously [47]. *C. thermocellum* cellulases were purified from cell-free broth harvested during stationary phase from a pH controlled reactor (Sartorius A+, Bohemia, NY) with initial substrate concentration of 5 g/L Avicel PH-105 (FMC BioPolymer, Philadelphia, PA, USA) using the optimized affinity digestion method as described previously [48]. Alternatively, cell-free broth was concentrated in a Biomax tangential flow filter with a NMWL of 10 kDa (Millipore, Billerica, MA, USA) and dialyzed in water overnight at 4 °C (Spectra/Por 6–8 kDa MWCO, Rancho Dominguez, CA, USA).

### Solubilization in serum bottles

Dry substrate was weighed out (solids content required to give 0.25 g glucan per bottle unless otherwise indicated), rehydrated with media (50 ml final volume), sealed and purged with 20 × 45 s of alternating cycles of vacuum and nitrogen. Bottles were autoclaved at 121 °C for 30 min and allowed to cool before remaining media components were added. Bottles were allowed to reduce for at least 1 h before inoculation with either enzymes or microbes. For microbial solubilization experiments, inoculum (2 % v/v) was taken from 2-day-old cultures with the same substrate, which is when the culture was most active (see Additional file 1: Figure S3). Cultures were incubated at the temperatures indicated above, with shaking at 180 rpm. Residual solids were collected from serum bottles (50 ml working volume) washed and centrifuged twice at 3000*×g* for 10 min before drying at 60 °C overnight and weighing.

### Bioreactor fermentations

Dry substrate was weighed out, rehydrated with water by purging with 20 × 45 s of alternating cycles of vacuum and nitrogen, transferred to reactor vessel, and autoclaved at 121 °C for 60 min. Reactor was purged with nitrogen, agitation (200 rpm) and temperature control was activated, and remaining media components were added. Once temperature and pH stabilized, a sample was removed to verify pH calibration and pH control was activated (setpoint of 7.0). Just prior to inoculation, the nitrogen purge was stopped as it can lead to stripping of CO2 and increased lag times. The exhaust was piped through a water lock to discourage infiltration of oxygen. Inoculum (2 % v/v) was taken from 2-day-old cultures as described above. Residual solids were centrifuged at 14,300×*g* for 15 min, the total wet weight was recorded, and sub-samples were taken to determine moisture content (average 13 weight  % solids).

### Substrates

Switchgrass (*Panicum virgatum*, Cave in Rock) was harvested either in June 2012 (mid-season, 100 % elongation, 35 % glucan, 27 % xylan, and 3 % arabinan after washing) or April 2013 (senescent, 42 % glucan, 26 % xylan, and 3 % arabinan after washing) at Rock Springs Research Farm (Spring Mills, PA). Further studies were conducted with switchgrass (*Panicum virgatum*, Alamo) harvested at University of Tennessee in October 2013 (38 % glucan, 27 % xylan, and 3 % arabinan after washing). *Populus tremuloides* (44 % glucan and 17 % xylan after washing) was harvested as described previously [49]. Feedstocks were washed as previously described [50] to remove soluble sugars, dried, and milled in either a ED-5 Wiley Mill (0.5–6 mm screens, Thomas Scientific, Swedesboro, NJ) or ZM200 centrifugal milling machine (0.2 screen, Retsch, Haan, Germany). Unless otherwise noted, substrate was loaded based on equal glucan content (5 g/L) and milled to pass through a 4-mm (switchgrass) or 0.5-mm (*Populus*) sieve. These were chosen as the largest particles sizes which reliably gave solubilization above that for uninoculated controls.

### Quantification of solubilization

Cell-free supernatants were analyzed by HPLC (Waters, Milford MA, USA) with an eluent of 5 mM sulfuric acid on an Aminex HPX-87H column (Bio-Rad, Hercules CA, USA) and detection by refractive index. Retention times of standards with typical hydrolysis and fermentations products were used to identify peaks in the chromatograms. Polymeric sugars were analyzed after acid hydrolysis as described previously [5] and detected as monomers by HPLC and corrected for sugar degradation. Feedstock and residual substrate composition was determined by quantitative saccharification as described by Saeman [51] and adapted by Sluiter [52], except the protocol was scaled down to use 0.1 g dry sample, 1 mL 72 % sulfuric acid, and 28 mL water. Hydrolyzed sugars were quantified by HPLC and corrected for sugar degradation. The total grams of non-carbohydrates were calculated by subtracting the total grams of carbohydrate (glucan, xylan, araninan) from the total grams of solids. Percent solubilization was calculated based on the following equation (for glucan solubilization):

$$\% {\text{glucan}}\;{\text{solubilization}}\; = \;\frac{{{\text{initial }}\;{\text{g }}\;{\text{glucan}} - \text{final}\;{\text{g}}\;{\text{glucan }}}}{{{\text{initial}}\;{\text{g}}\;{\text{glucan}}}}\; \times \;100$$

### Cotreatment

Solids (~25 g wet, average 13 weight  % solids) were ball milled in batches for 5 min (SFM-3, MTI Corporation, Richmond, CA with 15 11 mm steel balls) either before or after one 5-day fermentation of senescent switchgrass (Alamo) in a 2 L pH controlled reactor (Sartorius A+). Residual solids were washed and centrifuged twice at 14,300×*g* for 15 min and triplicate samples (unmilled or milled) were taken and dried to determine moisture content. A second 5-day fermentation was started with carbohydrate loading identical to that at the end of the first fermentation and residuals were collected as described above and stored at 4 °C until further analysis. Particle size distribution was determined with a Malvern Mastersizer Hydro 2000G (Worcestershire, UK). Optical properties of wood flour (refractive index of 1.53, absorption of 0.1) and distilled water (refractive index of 1.33) were used for the sample and dispersant, respectively.

### Sample processing for microscopy

Switchgrass samples were processed using microwave processing as described previously [53]. Briefly, samples were fixed 2 × 6 min (with variable power) in 2.5 % (v/v) gluteraldehyde buffered in 0.1 M sodium cacodylate buffer (EMS, Hatfield, PS) under vacuum. The samples were dehydrated by treating with increasing concentrations of ethanol and heating in a Pelco microwave oven for 1 min at each dilution [i.e., 15, 30, 60, 90 % (v/v), and 3 × 100 % ethanol]. After dehydration, the samples were infiltrated with LR White resin (EMS, Hatfield, PA) by incubating at room temperature for several hours to overnight in increasing concentrations of resin [15, 30, 60, 90 % (v/v), 3 × 100 % resin, diluted in ethanol]. The samples were transferred to capsules and the resin polymerized by heating to 60 °C overnight.

### Confocal scanning laser microscopy (CSLM)

Semi-thin sectioned samples were positioned on glass microscope slides and stained with 0.1 % acriflavine in water. Images were captured using a 60X 1.4NA Plan Apo lenses on a Nikon C1 Plus microscope (Nikon, Tokyo, Japan), equipped with the Nikon C1 confocal system using the Argon tunable laser at 488 nm, and operated via Nikon’s EZ-C1 software.

### Transmission electron microscopy (TEM)

LR White embedded samples were sectioned to ~60 nm with a Diatome diamond knife on a Leica EM UTC ultramicrotome (Leica, Wetzlar, Germany). Sections were collected on 0.5 % (v/v) Formvar-coated slot grids (SPI Supplies, West Chester, PA). All grids were post-stained for 4 min. with 2 % (w/v) aqueous uranyl acetate and 2 min. with Reynolds lead citrate. Images were taken with a 4 mega-pixel Gatan UltraScan 1000 camera (Gatan, Pleasanton, CA) on a FEI Tecnai G2 20 Twin 200 kV LaB6 TEM (FEI, Hilsboro, OR).

## Additional file

10.1186/s13068-015-0412-y Supplemental information

## Abbreviation

SSF: simultaneous saccharification and fermentation
