# Supplementary material for: Biological lignocellulose solubilization: comparative evaluation of biocatalysts and enhancement via cotreatment
Source: Biotechnol Biofuels. 2016 Jan 12;9:8. doi: 10.1186/s13068-015-0412-y (PMC4709877; doi:10.1186/s13068-015-0412-y)
Supplement: Supplementary file 1 — 10.1186/s13068-015-0412-y Supplemental information [file 13068_2015_412_MOESM1_ESM.docx]

**Table S1.** Performance of biocatalysts used in the Lynd lab for this study in comparison with other labs and/or prior studies.

| Biocatalyst | Substrate | Time, Basis | % Solubilization^a^ | | Source |
| --- | --- | --- | --- | --- | --- |
|  |  |  | Lynd lab, this study | Other labs and/or prior studies |  |
| *C. thermocellum* | Avicel PH105 | 2 day  % glucan | 99.6+0.1 | ~100 | [1](#_ENREF_1), Figure 1 |
| *C. bescii* | Washed senescent switchgrass | 5 day  % dry weight | 19.2 + 2 | ~12  ~26 | [2](#_ENREF_2), Figure 3  ^[3](#_ENREF_3)^, Figure 1A |
| *C. clariflavum* | Washed senescent switchgrass | 5 day  % glucan | 46 + 2 | ~42 | [4](#_ENREF_4), Figure 4B |
| *C. cellulolyticum* | MN301 | 5 day  % glucan | 92.1 + 1 | ~ 90 | [5](#_ENREF_5), Figure 2 |
| SSF | AvicelPH105 | 5 day  % glucan | 55.2 + 2 | 55 + 2 | Wyman lab |

^a^ The purpose of the comparisons made in this table was to test whether the solubilization data obtained in this study by the Lynd lab was similar to what has been reported in other labs and/or prior studies. For work with *C. besci, C. cellulolyuticumI, and SSF,* we used the same growth medium in this study as was used in the prior studies cited. In the case of *C. thermocellum* and *C. clariflavum,* we tested solubilization in both the rich medium used in prior studies and also a low carbon medium with small modifications as noted in the methods section, for which we observed higher solubilization. “Lynd lab” and “Wyman lab” refer respectively to the labs led by coauthors Lee Lynd at Dartmouth and Charles Wyman at University of California, Riverside

**Table S2**. Summary of porosity and volume-mean particle size of residual solids from *C. thermocellum* fermentation of washed senescent switchgrass with brief ball milling (5 min) either before or between two 5-day fermentations

|  |  | Pore Volume | Volume - Mean Particle Size |
| --- | --- | --- | --- |
|  |  | 20-200A | d (0.5) |
| No Milling | initial substrate | 0.13 |  |
|  | after 1st fermentation | 0.21 | 492.841 |
|  | after 2nd fermentation | 0.26 | 410.92 |
| Milling Before | initial substrate | 0.13 |  |
|  | after ball milling | 0.25 | 80.484 |
|  | after 1st fermentation | 0.36 | 88.4545 |
|  | after 2nd fermentation | 0.32 | 89.211 |
| Milling Between | initial substrate | 0.13 |  |
|  | after 1st fermentation | 0.21 | 492.841 |
|  | after ball milling | 0.25 | 36.4465 |
|  | after 2nd fermentation | 0.33 | 29.132 |


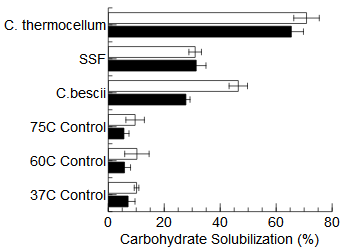


**Fig S1.**  Carbohydrate solubilization from washed mid-season switchgrass with an initial solids concentration of 13 g/L (black bars) or 2.5 g/L (white bars) after 5 days (5 g/L glucan and 1 g/L glucan, respectively). Fungal cellulase loading was 4.5 mg Ctec2/ g solid and 0.5 mg Htec2 / g solid. Uninoculated controls for each incubation temperature were analyzed to account for non-biological solubilization. Results are expressed as mean ± SD (n≥2)


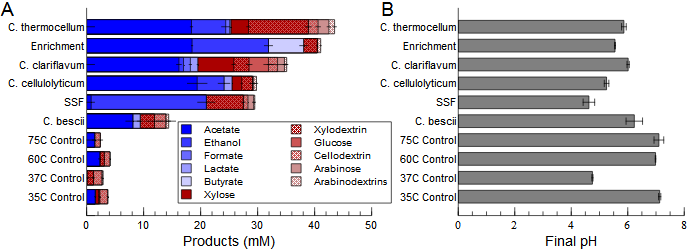


**Fig. S2.**  Characterization of supernatants after 5 day fermentation of washed mid-season switchgrass (4mm particle size) by various by various bacteria (2% inoculum) or SSF with yeast and fungal cellulase (4.5 mg Ctec2/ g solid and 0.5 mg Htec2 / g solid). A) Final concentrations of fermentation and hydrolysis products; and (B) final pH. Enrichment was selected on Avicel from horse manure compost. Results are expressed as mean ± SD (n≥2)


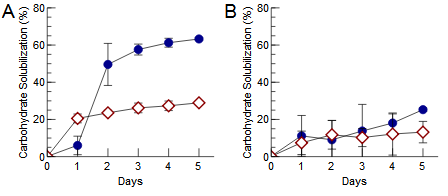


**Fig. S3.** Carbohydrate solubilization by *C. thermocellum* (2% inoculum) (filled circles) or fungal cellulase and yeast (open diamonds). Fungal cellulase loading was 4.5 mg Ctec2/ g solid and 0.5 mg Htec2 / g solid. Solubilization of uninoculated switchgrass (5 g/L glucan, 13 g/L solid, <4mm particle size) and *Populus* (5 g/L glucan, 11 g/L solid, <0.5mm particle size) was less than 10%. Results are expressed as means ± SD (n≥2). (A) Solubilization of washed mid-season switchgrass. (B) Solubilization of washed *Populus.*

**
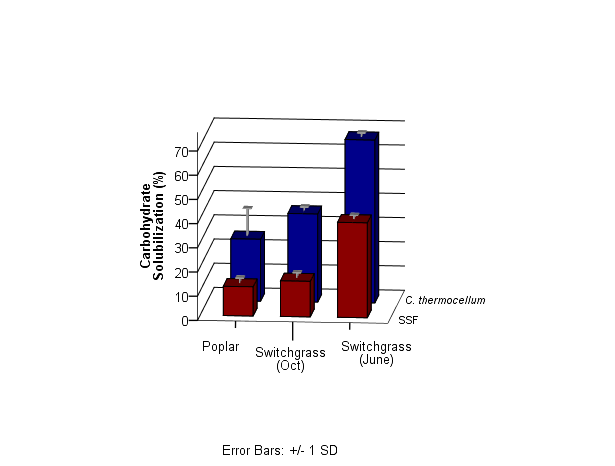
**

**Fig. S4.** Carbohydrate solubilization from washed *Populus* or switchgrass (harvest in June or Oct) (5 g glucan/L, 0.5mm particle size) by *C. thermocellum* (2% inoculum) or SSF (4.5 mg Ctec2/ g solid and 0.5 mg Htec2 / g solid) after 5 days. Data are reported as means ± SD.

**
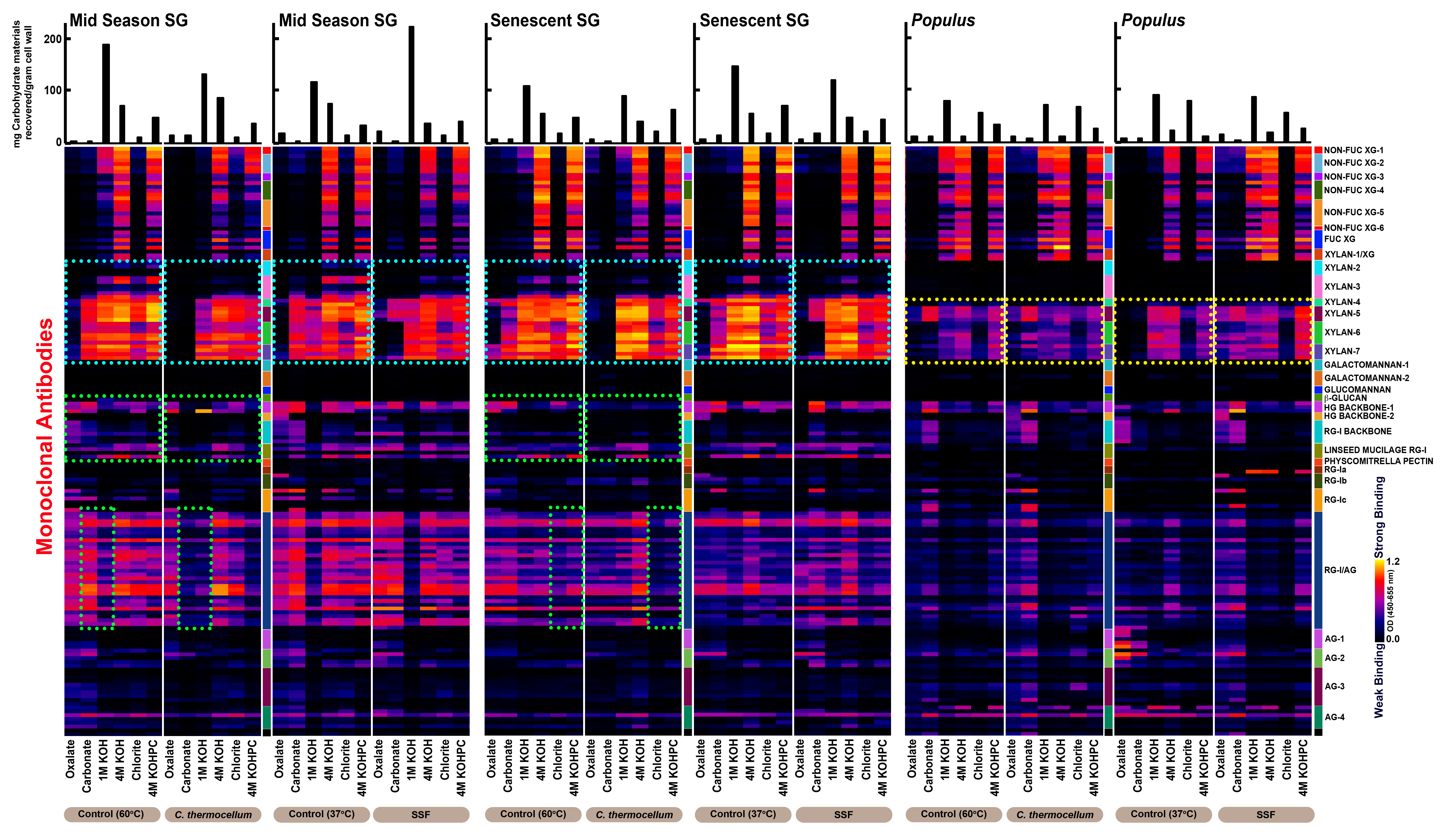
**

**Fig. S5.**  Glycome profiling of switchgrass (SG) and poplar biomass residues after incubation in the absence and presence of *C. thermocellum* or fungal cellulase/yeast. Sequential extracts of cell walls isolated from various biomass residues were prepared using ammonium oxalate (Oxalate), sodium carbonate (Carbonate), potassium hydroxide (1M KOH, 4M KOH and 4M KOHPC) and acidic sodium chlorite (Chlorite) as explained in Materials and Methods. The cell wall extracts were ELISA screened using 155 mAbs directed against glycan epitopes encompassing most major plant cell wall glycans. The resulting binding response data are represented as heatmaps with a bright yellow-red-black scale indicating the strength of the ELISA signal (bright yellow, red and black colors depict strong, medium, and no binding, respectively). The mAbs are grouped based on the cell wall glycans they principally recognize, as depicted in the panel at the right hand side of the figure. The actual amounts of material extracted from the cell walls at each extraction step are depicted as bar graphs at the top of the heatmaps. Blue and yellow outlined boxes highlight changes in xylan components of the walls resulting from incubation with the microbe or enzymes. Green outlined boxes highlight changes in the pectin and pectic arabinogalactan components of the cell walls.


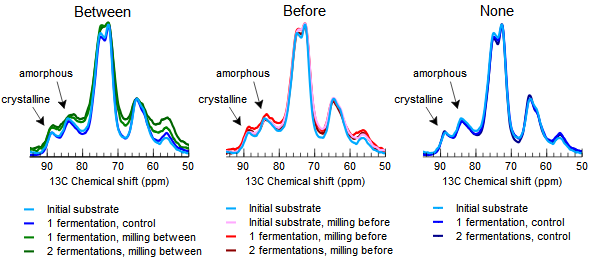


**Fig. S6.**  ^13^C solid-state magic-angle nuclear magnetic resonance spectra of fermentation residues. Changes in cellulose crystallinity were determined to be minimal based on the ratios of the intensities of the resonances for carbon nuclei in the interior crystalline cellulose regions (89 ppm) and carbon nuclei in amorphous or disordered regions (84 ppm).

**Materials and Methods**

**Glycome Profiling of biomass residues:** Sequential cell wall extractions and glycome profiling of various biomass residues were carried out as described previously [^6^](#_ENREF_6), [^7^](#_ENREF_7). Plant glycan-directed monoclonal antibodies (mAb) [^8^](#_ENREF_8) were from laboratory stocks (CCRC, JIM and MAC series) at the Complex Carbohydrate Research Center (available through CarboSource Services; http://www.carbosource.net) or were obtained from BioSupplies (Australia) (BG1, LAMP).

**Solid State NMR.** Solid-state NMR spectra, for determining cellulose crystallinity and lignin molecular weight estimates, were collected using high-resolution ^13^C cross-polarization/magic angle spinning (CP/MAS) with a Bruker Avance 200 MHz spectrometer (50.13 MHz) [^9^](#_ENREF_9). Samples were dried then packed in ZrO_2_ rotors and spun at 7000 Hz and all data was collected at ambient temperatures. A contact time of 2 ms with a 1 db ramp on the proton spin locking field was applied during cross polarization along with an acquisition time of 32.8 ms and a recycle delay of 1 s.

**NMR Cryoporometry.**  NMR Cryoporometry measurements were carried out as detailed [^10^](#_ENREF_10). The experiments were conducted on a Bruker Avance III 200MHz spectrometer. Spectra were collected using a 90° pulse and a 5s recycle delay. For each sample, 7-9g of hydrated material were loaded into a 10mm NMR tube. Samples were cooled to -45°C and the ^1^H NMR signal was recorded every 5°C, allowing 5 minutes for the temperature to equilibrate at each temperature, while heating up to 15°C. The intensity of the NMR signal is directly proportional to the fraction of unfrozen water in the sample. The pore volume can be calculated using the following equation [^10^](#_ENREF_10):

Pore volume (g water/g dry biomass) = IT*w/ (100*m)

Where IT is the signal intensity at a given temperature, w is wet sample weight and m is the dry sample weight. The pore volume from 20-200Å was calculated by summing the pore volumes from the temperature range of -35 to -5°C.

**Supplemental Appendix 1**

**Derivation of equations representing alternative hypotheses regarding the fate of the non-carbohydrate fraction of lignocellulose during biologically-mediated solubilization:**

Define the mass of carbohydrate solubilized per mass original solids, $s_{TS}^{C}$, by

$s_{TS}^{C}$ = $\frac{C\mathrm{solubilized}}{TS(0)}$=$\frac{c \mathrm{solubilized}}{C(0)}\frac{C(0)}{TS(0)}=$ $s_{C(0)}^{C}{}^{o}{f_{C}}$ [1]

where

$s_{j}^{i}$denotes solubilization of component i as a fraction of j, a function of time.

${}^{o}{f_{i}}$ denotes the mass ratio of component i to total solids at time = 0, a constant

*C* denotes mass carbohydrate

*TS* denotes mass total solids

Similarly, define the mass of non-carbohydrate solubilized per mass original solids, $s_{TS}^{NC}$, by

$s_{TS}^{NC}$ = $\frac{NC\mathrm{solubilized}}{TS(0)}$=$\frac{NC \mathrm{solubilized}}{NC(0)}\frac{NC(0)}{TS(0)}=$ $s_{NC(0)}^{NC}{}^{o}{f_{NC}}$ [2]

The mass of carbohydrate per mass original solids remaining at any given time is given by

$m_{C}=(1-s_{C\left( 0 \right)}^{C}) {}^{o}{f_{C}}$ [3]

and the mass of non-carbohydrate per mass original solids remaining at any given time is given by

$m_{NC}=(1-s_{NC\left( 0 \right)}^{NC}) {}^{o}{f_{NC}}$ [4]

Given [3] and [4], we can express the mass of carbohydrate per mass solids remaining at time t as

*f_c_* = $\frac{m_{c}}{m_{c}+m_{NC}}$ $=\frac{(1-s_{C\left( 0 \right)}^{C}) {}^{o}{f_{C}}}{\left( 1-s_{C\left( 0 \right)}^{C} \right){}^{o}{f_{C}}+(1-s_{NC\left( 0 \right)}^{NC}) {}^{o}{f_{NC}}}$ [5]

We have two cases:

a) $s_{NC(0)}^{NC}=0$ (inert non-carbohydrate)

For this case, equation [5] becomes

$f_{c}=\frac{(1-s_{C\left( 0 \right)}^{C}) {}^{o}{f_{C}}}{\left( 1-s_{C\left( 0 \right)}^{C} \right){}^{o}{f_{C}}+ {}^{o}{f_{NC}}}$

but since

${}^{o}{f_{NC}}=1- {}^{o}{f_{C}}$

the equation can be rewritten as

$f_{c}=\frac{(1-s_{C\left( 0 \right)}^{C})}{\frac{1}{{}^{o}{f_{C}}} - s_{C\left( 0 \right)}^{C}}$ [5a]

b) $s_{NC\left( 0 \right)}^{NC}= s_{C\left( 0 \right)}^{C}$ (“peeling hypothesis”)

For this case, equation [5] becomes

$f_{c}=\frac{(1-s_{C\left( 0 \right)}^{C}) {}^{o}{f_{C}}}{\left( 1-s_{C\left( 0 \right)}^{C} \right){}^{o}{f_{C}}+(1-s_{C\left( 0 \right)}^{C}) {}^{o}{f_{NC}}}=\frac{{}^{o}{f_{C}}}{{}^{o}{f_{C}}+ {}^{o}{f_{NC}}}= {}^{o}{f_{C}}$ [5b]

**Supplemental Appendix 2**

**Glycome Profiling Analyses of Biomass Residues:**

Glycome profiling was conducted on plant biomass residues originating from mid-season switchgrass (SG), late season switchgrass and poplar wood that were incubated either in the presence or absence of *C. thermocellum* and fungal cellulose and yeast, as described in Materials and Methods in the manuscript. The purpose of glycome profiling analyses was to delineate the changes in the overall composition and extractability of most major non-cellulosic cell wall glycans in these wall residues before and after incubation with these microbes/enzymes. Figure S5 depicts the data obtained from the glycome profiling analyses of all biomass residues studied. Overall, the glycome profiles of all control (control 60^o^C and control 37^o^C) mid-season and senescent switchgrass residues were similar. As expected and agreeing with the previous reports [^6^](#_ENREF_6) , the glycome profiles of all poplar control residues significantly differed from those of switchgrass control residues especially in that poplar extracts did not contain the xylan epitopes that are recognized bythe xylan-3 group of mAbs and, in general, lower amounts of extractable carbohydrates were recovered from poplar residues, especially in the 1M KOH extracts (see bar graphs in top panel).

Results were further analyzed in order to understand the plant biomass utilization by *C.thermocellum* and/or SSF with fungal cellulase and yeast. Significant differences were noted in the glycome profiles of both mid-season and senescent switchgrass residues that were incubated with *C.thermocellum* in comparison to the profiles of the respective untreated controls (control 60^o^C). In both *C.thermocellum* incubated mid-season and senescent switchgrass residues, a total disappearance of all xylan epitopes recognized by all xylan-specific groups of mAbs (xylan-3 through 7) was observed in the less harsh oxalate and carbonate extracts (see light blue dotted boxes, Fig. S5). Also, the amounts of extractable carbohydrate material were lower in the 1M KOH extracts in *C.thermocellum* incubated mid-season and senescent switchgrass residues. Overall, these data indicate that *C.thermocellum,* during its growth on switchgrass biomass, selectively solubilizes easily extractable xylans. Differences were also noted in the abundance of pectic components in the sequential extracts from *C.thermocellum* incubated mid-season and senescent switchgrass residues. A complete removal of rhamnogalacturonan back bone epitopes was observed in all extracts of *C.thermocellum* incubated mid-season switchgrass residues (see light green dotted boxes) and a complete removal of homogalacturonan epitopes was observed in extracts of *C.thermocellum* incubated senescent switchgrass residues (see light green dotted boxes, Fig. S5). Further, in *C.thermocellum* incubated mid-season switchgrass residues, a significant reduction in the abundance of pectic arabinogalactan epitopes (recognized by RG-I/AG groups of mAbs) were observed in the carbonate and 1M KOH extracts. However, in *C.thermocellum* incubated senescent swtichgrass residues, such a reduction in the abundance of pectic arabinogalactan epitopes was noted in chlorite and 4M KOHPC extracts (see light green dotted boxes, Fig. S5). Together, these data suggest that *C.thermocellum* specifically solubilizes loosely integrated xylans (both unsubstituted and substituted) and pectic glycans during its growth on switchgrass biomass.

Glycome profiles were also prepared for both mid-season and senescent switchgrass residues that had been incubated with fungal cellulase and yeast in comparison to their respective untreated controls (control 37^o^C). Incubation with fungal cellulase and yeast resulted in the removal of only the xylan-6 and xylan-7 epitopes from the carbonate extracts, while oxalate extracts remained unchanged (Fig. S5). Further, there was no reduction in the amounts of 1M KOH extractable carbohydrate in the case of fungal cellulase and yeast incubated mid-season switchgrass; rather a significant enhancement was observed. However, in the case of senescent switchgrass residues incubated with fungal cellulase and yeast, such a significant increase in 1M KOH extractable material was not observed. No major conclusive changes were noted in pectic components for either mid-season or senescent switchgrass residues that had been incubated with fungal cellulase and yeast. Overall, the data suggest that switchgrass xylan and pectin solubilization by fungal cellulase and yeast is not as efficient as in the case of digestion with *C.thermocellum.*

Glycome profiling analyses of poplar residues revealed that, in comparison to switchgrass, considerably fewer changes are brought about by incubation with either *C.thermocellum* or fungal cellulase and yeast. An enhanced extractability of xylan epitopes was observed in the oxalate and carbonate extracts for poplar biomass that had been incubated with either *C.thermocellum* or fungal cellulase and yeast (see yellow dotted boxes, Fig. S5). An enhanced abundance of pectic backbone epitope was noted in the carbonate extract of poplar biomass incubated with fungal cellulase and yeast. Also, enhanced xyloglucans extractability was noted in the 1M KOH extract of poplar biomass incubated with fungal cellulase/yeast. But, relative to switchgrass residues, significantly fewer and less dramatic changes were observed in the glycome profiles of poplar biomass that had been incubated with either *C.thermocellum* or fungal cellulase and yeast. These results suggest that solubilization of the more recalcitrant poplar cell walls by either *C.thermocellum* or fungal cellulase and yeast is less efficient when compared with the effects of these two systems on mid-season and senescent switchgrass residues.

References:

1. X. J. Shao, M. J. Jin, A. Guseva, C. G. Liu, V. Balan, D. Hogsett, B. E. Dale and L. Lynd, *Bioresource Technol*, 2011, 102, 8040-8045.

2. S. J. Yang, I. Kataeva, S. D. Hamilton-Brehm, N. L. Engle, T. J. Tschaplinski, C. Doeppke, M. Davis, J. Westpheling and M. W. W. Adams, *Appl Environ Microb*, 2009, 75, 4762-4769.

3. I. Kataeva, M. B. Foston, S. J. Yang, S. Pattathil, A. K. Biswal, F. L. Poole, M. Basen, A. M. Rhaesa, T. P. Thomas, P. Azadi, V. Olman, T. D. Saffold, K. E. Mohler, D. L. Lewis, C. Doeppke, Y. N. Zeng, T. J. Tschaplinski, W. S. York, M. Davis, D. Mohnen, Y. Xu, A. J. Ragauskas, S. Y. Ding, R. M. Kelly, M. G. Hahn and M. W. W. Adams, *Energ Environ Sci*, 2013, 6, 2186-2195.

4. J. A. Izquierdo, S. Pattathil, A. Guseva, M. G. Hahn and L. R. Lynd, *Biotechnol Biofuels*, 2014, 7, 136.

5. M. Desvaux, E. Guedon and H. Petitdemange, *Appl Environ Microbiol*, 2000, 66, 2461-2470.

6. J. D. DeMartini, S. Pattathil, U. Avci, K. Szekalski, K. Mazumder, M. G. Hahn and C. E. Wyman, *Energ Environ Sci*, 2011, 4, 4332-4339.

7. S. Pattathil, U. Avci, J. S. Miller and M. G. Hahn, *Methods Mol Biol*, 2012, 908, 61-72.

8. S. Pattathil, U. Avci, D. Baldwin, A. G. Swennes, J. A. McGill, Z. Popper, T. Bootten, A. Albert, R. H. Davis, C. Chennareddy, R. H. Dong, B. O'Shea, R. Rossi, C. Leoff, G. Freshour, R. Narra, M. O'Neil, W. S. York and M. G. Hahn, *Plant Physiol*, 2010, 153, 514-525.

9. M. F. Davis, H. A. Schroeder and G. E. Maciel, *Holzforschung*, 1994, 48, 186-192.

10. C. I. Ishizawa, M. F. Davis, D. F. Schell and D. K. Johnson, *J Agr Food Chem*, 2007, 55, 2575-2581.
